# Volcanic ash melting under conditions relevant to ash turbine interactions

**DOI:** 10.1038/ncomms10795

**Published:** 2016-03-02

**Authors:** Wenjia Song, Yan Lavallée, Kai-Uwe Hess, Ulrich Kueppers, Corrado Cimarelli, Donald B. Dingwell

**Affiliations:** 1 Department of Earth and Environmental Sciences, Ludwig-Maximilians-Universität (LMU) Munich, Theresienstrasse 41/III, 80333 Munich, Germany; 2 Department of Earth, Ocean and Ecological Sciences, University of Liverpool, Liverpool L69 3GP, UK

## Abstract

The ingestion of volcanic ash by jet engines is widely recognized as a potentially fatal hazard for aircraft operation. The high temperatures (1,200–2,000 °C) typical of jet engines exacerbate the impact of ash by provoking its melting and sticking to turbine parts. Estimation of this potential hazard is complicated by the fact that chemical composition, which affects the temperature at which volcanic ash becomes liquid, can vary widely amongst volcanoes. Here, based on experiments, we parameterize ash behaviour and develop a model to predict melting and sticking conditions for its global compositional range. The results of our experiments confirm that the common use of sand or dust proxy is wholly inadequate for the prediction of the behaviour of volcanic ash, leading to overestimates of sticking temperature and thus severe underestimates of the thermal hazard. Our model can be used to assess the deposition probability of volcanic ash in jet engines.

## Abstract (web summary)

Volcanic ash is hazardous to jet engines, with high temperatures in turbines causing ash particles to melt and stick to the engine, adversely affecting turbine function. Here, the authors explore the spectrum of natural ash compositions and their behaviour and impact at high temperatures.

## Graphical abstract

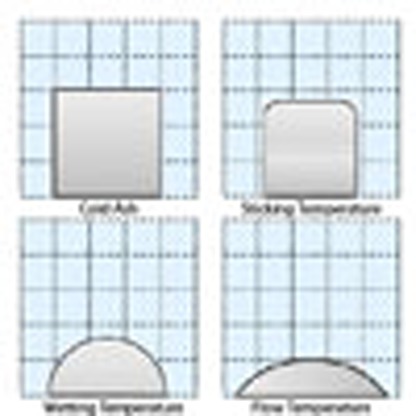

Safe air travel activity requires clean flight corridors^1^. But in Earth's atmosphere, particles (sand from desert storms, incinerated residues lofted into clouds and volcanic ash erupted at volcanoes) are often present and their concentrations must be carefully monitored through initiatives such as the International Airways Volcano Watch created by the International Civil Aviation Organization^2^,^3^,^4^. Particles present in the atmosphere, whether volcanic ash, dust or sand, may present a critical risk to aviation safety^5^,^6^. When these particles are ingested into jet engines, whose interiors (for example, the combustor and turbine blades) reach 1,200–2,000 °C (refs 7, 8, 9), they can abrade, melt and stick to the internal components of the engine, clogging ventilation traps of the cooling system as well as imparting substantial damage and potentially resulting in catastrophic system failure^10^,^11^,^12^,^13^,^14^. As air traffic grows and airspace corridors expand worldwide, the potential threat of sand, dust and volcanic ash clouds increases, and efforts clearly must be made to identify the potential danger in real time^15^ and to mitigate the risk^16^.

Our current understanding of particle interaction with jet engines relies exclusively on outdated tests using dust and sand particles^17^,^18^,^19^,^20^. Early studies on sand were prompted by the need to understand its impact on fuel efficiency, and on filter longevity^19^. The tests demonstrated that the investigated sands, primarily consisting of crystalline SiO_2_ (quartz), melt at very high temperatures (>1,700 °C) and, as such, it has long been advocated that flights may be permitted under low sand concentrations (<2 mg m^−3^) in the atmosphere^21^. Sand, however, significantly differs from the spectrum of mineralogical assemblages contained in volcanic ash^21^. As a result, the lack of understanding of volcanic ash behaviour at temperature above 1,100 °C resulted in a ‘zero ash tolerance' policy during the recent 2010 eruption at Eyjafjallajökull (Iceland), causing prolonged interruption in air traffic activity and considerable economic loss^22^. Here we provide a general description of volcanic ash behaviour at temperature up to 1,650 °C to ensure that hazard assessment models are improved and that future situations can therefore be mitigated more accurately.

Volcanic ash is volumetrically the most important product of explosive volcanism, and its presence in the Earth system can have significant and complex impacts, which we are only beginning to understand fully^23^. The silicate fraction of volcanic ash consists of juvenile fragments (vitric and crystalline) and pulverized rock (lithics) less than 2 mm in size^24^. The frequency of ash emission, and the uncertainties that still surround the mechanisms of ash emission, together with the widespread dispersal and prolonged residence time of ash at high altitudes in the atmosphere, qualifies volcanic ash as a serious hazard that threatens jet operations worldwide and at all times^1^,^2^. The behaviour of volcanic ash within natural or anthropic environments is further complicated by the wide variability in the physical and chemical states of ash, which arises from different volcanic events across the globe, or even during individual eruptions at a single volcano. These complexities can potentially result in highly variable behaviour of ash on reheating^25^. Above the glass transition temperature, volcanic ash particles containing glass sinter—a process driven by surface tension forces and regulated by viscous relaxation of the melt^26^,^27^. These processes may also be accompanied by variable amounts of melting and crystallization of crystalline phases^26^. Rock melting near thermodynamic equilibrium conditions has been extensively investigated and is the basis of modern petrology^28^,^29^; however, volcanic ash ingested into a jet engine will be subjected to rapid heating, which may additionally shift the conditions of sintering and melting to higher temperatures under such dynamic disequilibrium conditions^27^,^30^. Reconnaissance studies have recently investigated the thermomechanical properties of volcanic ash erupted in 2010 at Eyjafjallajökull^21^ and in 2012 at Santa María^31^ volcanoes. During heating to jet engine operation temperatures, a complex melting process, variably involving elements of both shrinkage and swelling, due to a combination of sintering, melting, vesiculation, outgassing and viscous flow, has been observed to occur^31^. The published data to date however are insufficient to constrain the effect of these processes in the form of a generalized model. Similarly, much effort has been made to assess the impact of volcanic ash on turbine degradation and jet engine operation^32^,^33^; yet, the lack of a general characterization of the range of volcanic ash melting behaviour at high temperatures prevents generalization of the conclusions of those studies for a fundamental understanding of ash deposition behaviour in jet engines.

Here the fusion of natural volcanic ash is systematically described. We empirically demonstrate that a set of characteristic temperatures (defined below) serve well as an effective parameterization of the sticking potential and relative propensity for deposition of volcanic ash in jet engines. We experimentally constrain the conditions leading to ash deposition onto hot surfaces as a function of bulk chemistry, expressed here as an index of the ratio of basic to acidic major oxides (*R*_b/a_) and heating rate. Our findings and their comparison with the fusion characteristics of dust and sand standards, used by the International Organization for Standardization (ISO), reinforce the preliminary conclusion that volcanic ash behaviour cannot be approximated by that of dust or sand particles^21^. Doing so will lead to significant underestimation of the thermal hazards arising from volcanic ash–jet engine interactions and should be avoided.

## Results

### Volcanic ash fusion behaviour

**Figure 1:**
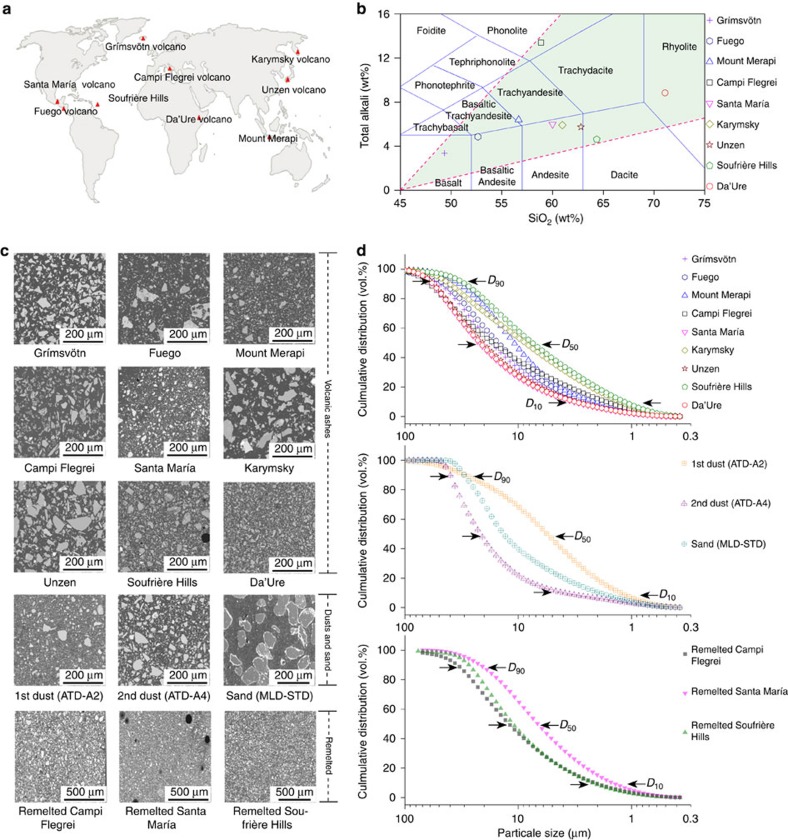
Physical and chemical characteristics of the samples tested. (**a**) Volcanoes from which the volcanic ash samples were selected around the world. (**b**) Geochemical classification of the ash samples, showing that the tested chemistries span the range of magma composition of the most common volcanic eruptions. (**c**) Scanning electron microscope images of embedded starting material of each sample tested. (**d**) Cumulative volume distribution of the samples tested. The *D*_10_, *D*_50_ and *D*_90_ values constrain the smallest particle size, which contribute to define the coarsest 10, 50 and 90% particle fraction from the cumulative volume distribution, respectively.

Volcanic ash samples were collected at nine volcanoes across the globe (Fig. 1a). The samples selected span the range of chemical variability commonly encountered in terrestrial explosive volcanism (Fig. 1b and Supplementary Table 1). To analyse quantitatively the fusion behaviour of volcanic ash at high temperatures, a standard procedure documented by ISO was employed. This procedure involves cylindrical volcanic ash compacts (3 mm diameter × 3 mm height) (1) synthesized by compressing loose ash at a fixed force in a die (Supplementary Fig. 1), (2) placed in a heating microscope and (3) subsequently heated to 1,650 °C at 10 °C min^−1^. During heating, geometrical changes are monitored and classified following standard procedures employed in energy engineering (Supplementary Figs 2–4 and Supplementary Data 1, see Methods for details, also see reconnaissance study of volcanic ash behaviour^31^). Application of this method to nine selected volcanic ash samples provides a robust first-order constraint on the compositional dependence of the thermal response of natural volcanic ash during reheating.

**Figure 2:**
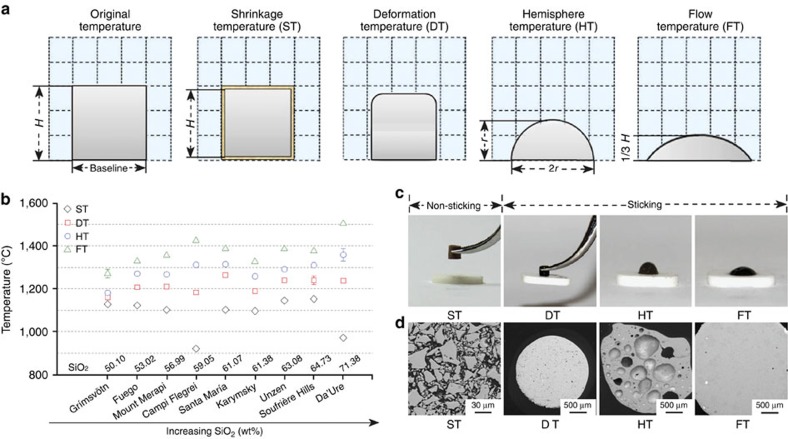
Geometric characterization of volcanic ash compacts at the characteristic temperatures. (**a**) Geometrical definition of four characteristic temperatures (ST, DT, HT and FT) in the volcanic ash melting process (see Methods and ref. 12). Each dotted box equates to an area of 1 mm × 1 mm. (**b**) Distribution of the four characteristic temperatures for the nine volcanic ash, ordered as a function of SiO_2_ content. Each data point represents the mean value of two independent measurements; the s.d.'s are illustrated and generally found to be smaller than the symbol. (**c**) Typical behaviour observed during a test, here with Grímsvötn's volcanic ash, along with (**d**) back-scattered electron images of corresponding microstructures of the volcanic ash at the four characteristic temperatures.

On heating, all of the volcanic ash compacts investigated underwent a systematic geometrical evolution, which may be categorized by temperatures corresponding to the attainment of four characteristic states: a shrinkage temperature (ST); a deformation temperature (DT); a hemispherical temperature (HT); and a flow temperature (FT) (Fig. 2a,b and Supplementary Table 2). Mechanically, the evolution of characteristic temperatures defines the ability of ash (1) to sinter to a coherent mass, ST; (2) to stick to surfaces due to melting, DT; (3) to spread and wet surfaces, HT; and (4) to flow significantly viscously under gravity, FT (Fig. 2c and Supplementary Fig. 5). This parameterization can be further employed to divide the thermal behaviour of volcanic ash into (a) a regime <ST for which ash does not stick and (b) a regime (ST≤DT), where ash initiates sticking, as well as (c) a regime ⩾DT, where ash sticks readily and macroscopically to surfaces due to particle melting and low melt viscosity (Fig. 2d). Importantly, the sticking potential of volcanic ash increases with temperature above DT as viscosity decreases. Henceforth, we use DT—a temperature objectively defined by a given sample geometry—as the threshold beyond which volcanic ash sticks onto hot surfaces. In what follows, this is the manner in which we (1) quantify the thermomechanical, and ultimately, the rheological behaviour of ash as it melts and (2) assess the sticking potential and deposition behaviour at high temperatures and as a function of heating rate.

**Figure 3:**
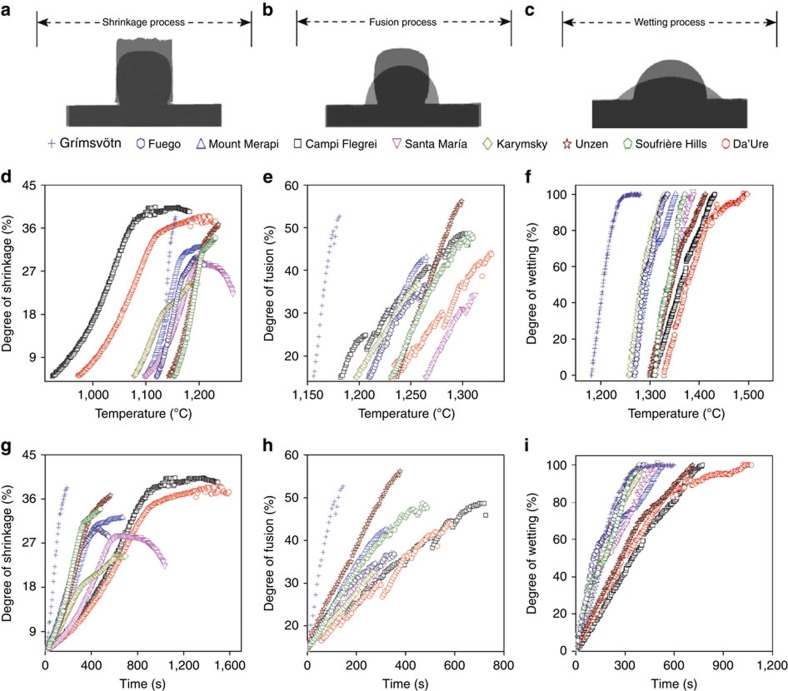
Geometrical evolution of volcanic ash compacts. Typical shape changes during heat through the stages of (**a**) shrinkage, (**b**) fusion and (**c**) wetting. In this example, the shape of volcanic ash compact from Fuego is shown. The morphological evolution is quantified as a function of (**d**–**f**) temperature and (**g**–**i**) time.

During heating, the sample geometry evolves distinctly at each stage (Fig. 3). Between ST and DT, the sample area diminishes as the samples shrink at rates that vary with temperature and through time (Fig. 3a,d,g). This results from densification during the formation and growth of neck between ash particles as they sinter (Supplementary Fig. 6). Between DT and HT, fusion causes a change in shape factor as the samples' sharp edges soften and corners become rounded, evolving to a half sphere (Fig. 3b,e,h) due to melting (Supplementary Fig. 6). Between HT and FT, the samples' base widens as it spreads onto, and wets, the substrate with increasing temperature and time (Fig. 3c,f,i).

### Thermal constraints on volcanic ash versus dust and sand

**Figure 4:**
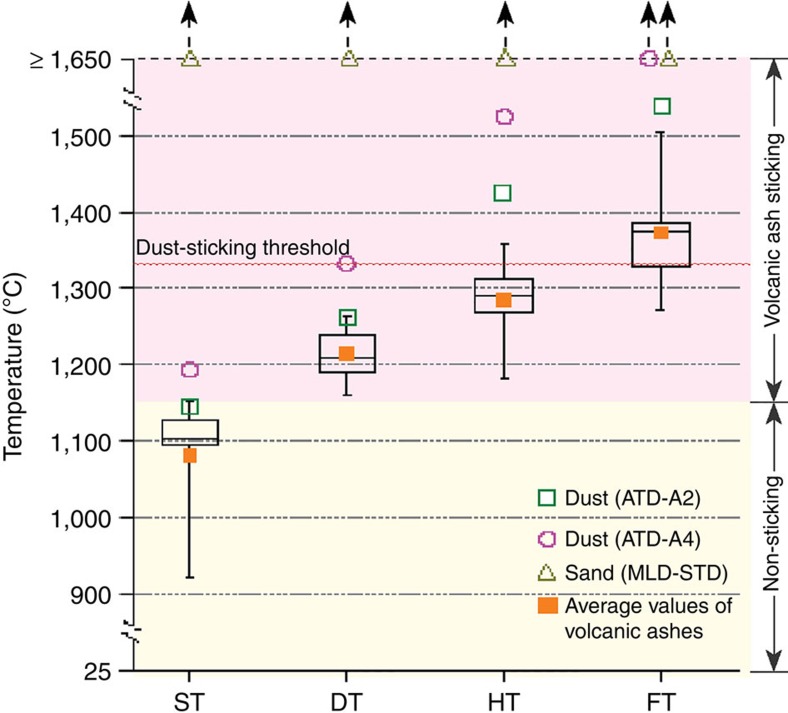
Thermal constraints on particle behaviour at high temperatures. The range of values of ST, DT, HT and FT of nine volcanic ash samples are represented in this box-and-whisker plot, together with those of the two standard dusts and one standard sand. The lower and upper sides of each box indicate the 25th and 75th percentiles, respectively. Inside the boxes, the solid lines indicate the median and the orange squares indicate the mean of the characteristic temperatures of the nine volcanic ash samples. Outside the boxes, small tick marks indicate minimum and maximum values. Note that some of the characteristic temperatures of sand and dust exceed the operational limit of our heating microscope (1,650 °C), yet these clearly supersede the low-temperature range of sticking ability of volcanic ash.

To compare the volcanic ash fusion behaviour with that of other particles potentially ingested into jet engines, we acquired three ISO standard materials used in engineering and military applications: (1) Arizona test dusts (ATD-A2 and ATD-A4); and (2) military standard test sand (MIL E 5007C; Fig. 1c)^21^. Comparison of the thermal characteristic temperatures obtained for volcanic ash with those for these dust and sand samples reveals a striking discrepancy (Fig. 4). Volcanic ash shares little similarity with the thermomechanical response of sand, and its similarity to dust is questionable at best. Whereas volcanic ash and the dust standard ATD-A2 display a similar sintering onset (ST) and sticking onset temperatures (DT), the subsequent corresponding wetting (HT) and flow (FT) temperatures differ by hundreds of degrees. This discrepancy increases further when comparing volcanic ash with sand and with a second dust standard ATD-A4. In fact, the fusion temperatures of these dust and sand samples are so high that they cannot be obtained using our present experimental set-up. These results therefore reinforce qualitatively the previous conclusion that the approximation of the thermomechanical behaviour of volcanic ash using sand or dust is wholly inadequate. Furthermore, the propensity for volcanic ash to interact with hot turbines at temperatures below those predicted by sand and dust behaviour can be quantified via the characteristic temperatures DT, HT and FT, which are overestimated in average by 92, 206 and 225 °C, respectively, based on dust particles, and by 436, 365 and 277 °C, respectively, based on sand.

### Physical and chemical controls on fusion behaviour

The considerable variability of the four characteristic temperatures between different volcanic samples, and their distinctness with respect to dust and sand, raises the general question of the effect of particle properties (size and shape), crystal fraction and chemical composition on fusion behaviour. For the samples tested herein, all particles are finer than 63 μm (as required for preparation of standard powder compacts for thermal optical examination in a heating microscope; Fig. 1d). Micro-textural analysis reveals that on heating through DT, individual volcanic ash grains become agglutinated (Fig. 2d), thus assign the potential effect of grain size to conditions where sintering dominates, that is, between ST and DT. The rate of densification by sintering decreases with increasing grain size, as individual grain curvature decreases, minimizing the effect of surface tension^26^. Thus, grain size may play a role for the onset of ash sticking at the lowest temperatures (that is, below DT), but when the ash temperature exceeds DT and begins to liquefy, its viscosity decrease leads to favouring of gravity (over surface tension) as a dominant force driving densification and flow. Therefore, we expect the role of the impact of grain size on the sticking potential of volcanic ash to decrease at the high temperatures experienced in jet engines.

**Figure 5:**
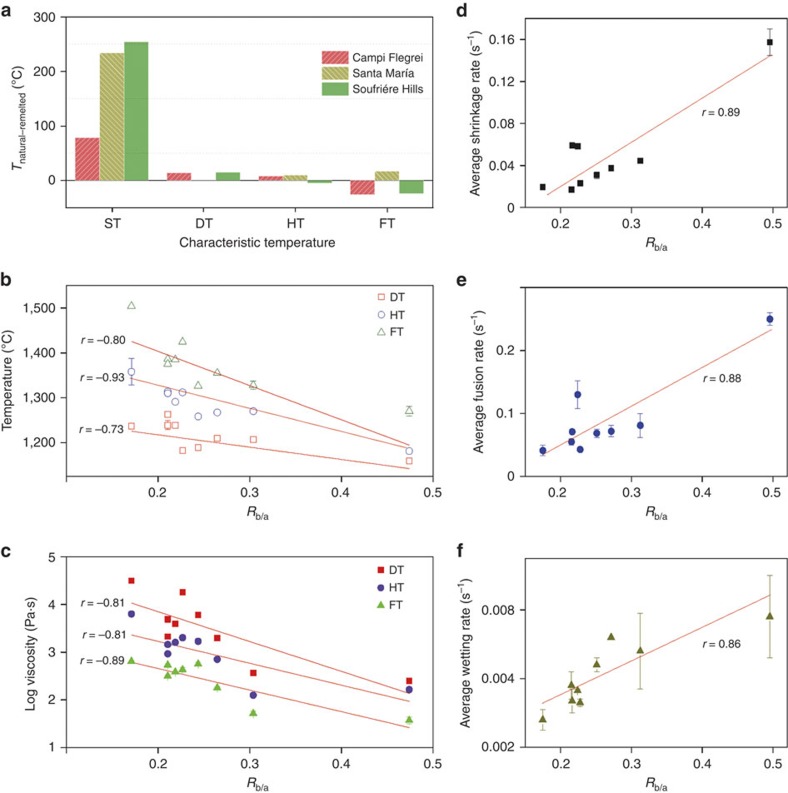
Chemical parameterization of volcanic ash fusion and rheology. (**a**) Net difference between the characteristic temperatures of three natural volcanic ash and that of three remelted ash from the same samples (*T*_natural−remelted_). (**b**) Chemical dependence of the characteristic temperatures using the ratio of acidic to basic major oxides (*R*_b/a_). The data show good linearity and the best fits yield the following regressions: DT*=*1,282–263 *R*_b/a_ (*r*=−0.73; *n*=9); HT=1,418–517 *R*_b/a_ (*r*=−0.93; *n*=9); and FT=1,556+762 *R*_b/a_ (*r*=−0.80; *n*=9). (**c**) Chemical dependence of the estimated viscosity of molten volcanic ash (*η*), as a function of *R*_b/a_. The data agree equally well with the geochemical composition of the ash and the best fits yield: for DT, log *η=*5.11–6.29 *R*_b/a_ (*r*=−0.81; *n*=9); for HT, log *η*=4.24–4.89 *R*_b/a_ (*r*=−0.81; *n*=9); and for FT, log *η*=3.58–4.59 *R*_b/a_ (*r*=−0.80; *n*=9) (**d**–**f**) Chemical composition dependence of sample geometry evolution. The data show that the average (**d**) shrinkage rate, (**e**) fusion rate and (**f**) wetting rate are linearly proportional to *R*_b/a_ (red line, linear fit through the data; *r*, correlation coefficient to indicate the accuracy of a regression). The values plotted represent the average of two independent experiments, and the s.d. of each sample is plotted.

Volcanic ash generally contains different fractions of crystals and glass. To test the effect of these phases, we remelted three natural volcanic ash samples (thereby excluding crystal content), then quenched, crushed and sieved them to the similar grain size fraction as their natural counterparts (Fig. 1d and Supplementary Fig. 7). We compared the behaviour of the remelted samples with that of chemically equivalent natural ash (Supplementary Table 1). We note that the presence of crystals primarily affects the sample evolution at low temperature, increasing ST by 79–254 °C (Fig. 5a). In contrast, DT, HT and FT vary by as little as 0–15, 5–10 and 17–26 °C, respectively. This important constraint suggests that the presence of crystals does not affect the behaviour of ash beyond DT—regime for which volcanic ash sticks readily onto hot surfaces.

After excluding initial mineralogy and grain size as primary controls on the geometrical response of ash samples above DT, we turned our attention to chemistry as a control on the characteristic temperatures of volcanic ash. The suite of volcanic ash samples tested is chemically diverse. In our tests we observe that SiO_2_-poor ash sticks and melts more readily than SiO_2_-rich ash; this makes volcanic ash more prone to interaction with jet engines than quartz sand. If chemistry is a primary control, then we should seek to constrain its effects on the temperature and viscosity of each sample as they undergo fusion through the four characteristic temperatures. To cope with the differing individual roles of the chemical components in dictating the fusion behaviour of these multicomponent volcanic ash samples, we parameterize the chemistry of each volcanic ash sample using the concept of an base–acid ratio of major oxides (*R*_b/a_), which compares the amount of basic oxides (CaO, FeO, MgO, K_2_O, Na_2_O and MnO) and acidic oxides (SiO_2_, Al_2_O_3_, TiO_2_ and P_2_O_5_; Fig. 5b and see Methods)—a standard index widely used to predict coal ash deposition in coal combustion^34^. We correlate *R*_b/a_ with the main characteristic temperatures, and the data show good linearity and the best fits arise the following regressions: DT=1,282−263*R*_b/a_ (*r*=−0.73; *n*=9), HT=1,418−517 *R*_b/a_ (*r*=−0.93; *n*=9) and FT=1,556+762 *R*_b/a_ (*r*=−0.80; *n*=9). We compare the power of *R*_b/a_ to constrain the temperature and viscosity of the molten ash at each characteristic temperature with that of other metrics such as the basicity index (CaO/SiO_2_)^35^, the number of non-bridging oxygens per tetrahedron (NBO/T)^36^ and SiO_2_ (Supplementary Fig. 8). The parameter *R*_b/a_ appears to provide the best correlation between chemistry and the main characteristic temperatures DT (sticking point) and HT, whereas the predictive power of SiO_2_ exceeds slightly that of *R*_b/a_ in constraining FT (the flowability); but as *R*_b/a_ remains accurate to describe all characteristic temperatures. Thus, we propose the use of *R*_b/a_ as a useful metric to simplify the description of multicomponent chemical system of natural volcanic ash.

Volatiles are an important control on explosive eruptions, as well as the physical properties of the eruptive materials and their presence in our samples deserves separate consideration. During the melting process of volcanic ash, a certain degree of vesiculation takes place as H_2_O (the dominant volatile in volcanic ash) solubility decreases and causes exsolution^37^,^38^. Water concentration, as well as the fraction of exsolved volatile in bubbles is also an important control on melt viscosity^39^. We have quantified the degree of water loss on heating and find that most of the water is liberated during heating to 600–700 °C (Supplementary Fig. 9), which is typical of molecular water adsorbed on the sample surface^37^. Beyond this point, we note very minor weight loss (<0.25 wt%) associated with water exsolution, which is completed by about 1,200 °C. However, observation of the microstructures of each sample at DT and ST (Supplementary Fig. 6) demonstrates that pores are present at DT and grow up to HT, inducing the observed minor inflation. Since most of the water is expelled in the first 600–700 °C of heating, it is very likely that the expansion of air trapped between grains after sintering, rather than the exsolution of dissolved water in matrix glasses, control the sample inflation between DT and FT^26^,^27^. Thus, for temperature conditions allowing ash to stick, an insignificant amount of water remains in the melt structures, thereby minimizing its impact on the rheology of molten ash. The rapid dehydration of volcanic ash during heating may explain why the major elements used in *R*_b/a_ suffice to constrain the characteristic temperatures.

The viscosity of the molten ash was estimated using the viscosity model of magmatic liquids developed by Giordano *et al*. - GRD calculator for characteristic temperatures (DT, HT and FT), based on the bulk chemistry.^25^ Again, for a given characteristic temperature, we find that viscosity generally decreases linearly with an increase in *R*_b/a_ (Fig. 5c). Thus, this distinction in both the temperature and the viscosity as a function of *R*_b/a_ suggests that bulk chemistry, and especially the fraction of major oxides, is a primary control on the rheological behaviour of volcanic ash as it melts. To ascertain whether viscous relaxation is the dominant control on the characteristic temperatures, we analyse the rate of morphological changes between each characteristic temperature (Fig. 5d–f). The analysis shows that the average rates of shrinkage (Fig. 5d), fusion (Fig. 5e) and wetting (Fig. 5f) scale linearly with the compositional parameter *R*_b/a_, which supports the conclusion that the viscosity of the bulk material controls the kinetic of volcanic ash melting, sticking and flow in jet engines.

### Kinetic effects on volcanic ash melting

**Figure 6:**
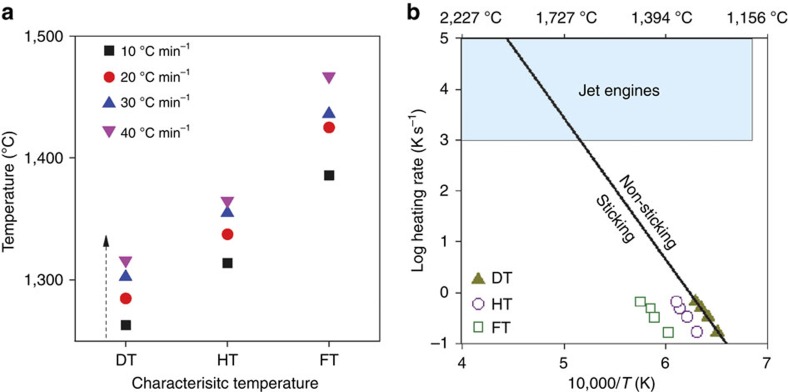
Heating rate effects on volcanic ash fusion. (**a**) Mean temperature values of DT, HT and FT of Santa María volcanic ash as function of heating rate. (**b**) Thermokinetic control on the characteristic temperatures of molten ash. The line is the Arrhenian regression that best fits temperature (T) at DT, as a function of heating rate (*q*): log (*q*)=17.325+(−5.32 × 10^5^)/(19.144·*T*) (*r*=0.99; *n*=4). Extrapolation to jet engine conditions (blue box) defines the temperature and heating rate window for which ash may melt and stick to jet engines.

Particles ingested in jet engine are subjected to very high temperatures (1,200–2,000 °C) for very short durations (<1 s)^40^. During their course through this extreme environment, particles are heated at very high rates (of several thousands of °C s^−1^)^41^, which may generate a dynamic influence on fusion and sticking behaviour. To simulate such dynamics and their potential kinetic consequences we tested the Santa María volcanic ash at different, controlled heating rates to be able to extrapolate our findings to conditions aiming to simulate jet engine operation. We tested four heating rates (10, 20, 30, 40 °C min^−1^), as higher heating rates cannot be achieved without significant loss of accuracy with the current experimental set-up. These tests demonstrate how the main characteristic temperatures (DT, HT and FT) increase with heating rate (Fig. 6a). When plotted as a function of heating rate, the characteristic temperatures demonstrate a remarkable linearity (Fig. 6b). We assess the conditions for ash melting in jet engines by extrapolating the data set at DT using an Arrhenian regression. The best fit of the threshold temperature of ash sticking, *T* (in K), as a function of heating rate (*q* in K s^−1^) is resolved to log(*q*)=*A*+(−*B*·1,000)/(ln10·*R*·*T*), where *A* is a pre-exponential constant constrained at 17.325 K s^−1^, *B* is the activation energy constrained at 531.96 kJ mol^−1^ and *R* is the gas constant (*r*=0.99; *n*=4). Just as DT varies between ash sample, the relationship between DT and heating rate varies equally owing to different compositions, mineralogical assemblages and dynamic of the fusion process; yet, due the fact that DT is achieved when the ash is mostly molten and that it only varies slightly between ash samples, we suggest that this rate-dependent regression may be used as a first-order approximation of ash fusion behaviour. Using this we can estimate the temperature and heating rate window for which different ash may melt and stick in jet engines. We conclude that knowledge of (1) volcanic ash chemistry, (2) its rate-dependent sticking threshold temperature and (3) the heating rate conditions is necessary and sufficient to assess its impact when ingested into jet engines.

## Discussion

The volcanic ash deposition process in a jet turbine is potentially complex^42^,^43^. Volcanic ash in the air stream enters the inner liners of the combustors and completely or partially melts under the flame (around 2,000 °C), at which point part of the ash deposits in the combustor fuel nozzle^11^,^44^. Molten volcanic ash particles within high-energy airflow escape the combustor to enter the turbine and impact the stationary (for example, inlet nozzle guide vanes) and rotating airfoils (for example, first-stage high-pressure turbine blades) at high speed (up to Mach 1.25) in different directions, with the result that ash may stick, flow and remain liquid or solidify^40^.

In modern aircrafts, jet engines operate at significantly higher gas temperatures than their predecessors to satisfy the ever-increasing demand for fuel efficiency. To mitigate such extreme conditions the metal parts in the hottest regions of gas-turbine engines are protected by thermal-barrier coatings (TBCs) made of refractory-oxide ceramic of 7–8 wt% Y_2_O_3_-stabilized ZrO_2_ (7YSZ)^45^,^46^. When volcanic ash deposits onto the surface of TBCs, dissolution reactions take place and as the TBCs degrade, element diffusion and incorporation of TBC fragments chemically differentiate the melt^47^. Through this reaction the TBCs deteriorate prematurely, exposing the more reactive metal surface, which allows interactions with hot gases and molten silicate melts to accelerate. Further degradation can be caused as TBCs spall off due to differential cooling contraction when jet engines stop.

In our experiments volcanic ash compact undergoes fusion on a dense, alumina oxide substrate. This material chemically and texturally differs from TBCs used in jet engines as such we remain cautious when employing our findings to constrain the full behaviour of volcanic ash ingested in jet engines. Despite the fact that contamination of the molten ash due to reaction with the TBCs may affect the rheology of the melt and the efficacy of wetting and corrosion, the type of coating or substrate material does not likely influence the deposition of volcanic ash as the particles only stick if they are already molten on contact with a surface. In such a case, our results suggest that the chemical composition and the thermal path are the most important controls on volcanic ash interaction with jet engines.

This study quantifies how easily volcanic ash particles may be expected to begin to interact with the hot parts of jet engines. The systematic description of volcanic ash and its comparison with dust and sand ISO standards support the conclusion that the bulk chemistry of volcanic ash is the dominant control on its ability to melt, stick and flow. The results further demonstrate that the assumption that volcanic ash interaction can be approximated by that of dust or sand particles can lead to significant errors as volcanic ash melt and remobilize at far lower temperatures than dust or sand. Thus, any robust future model to assess quantitatively the risk of volcanic ash interaction with jet engines must first be based on chemistry and resultant melt rheology. As a next step, we surmise that other important factors such as the effects of ingested ash concentration and residence time deserve equal consideration to understand the scale of damage during an aircraft encounter with an ash cloud. To further close the gap to the development of reliable mitigation protocols for ash–jet turbine interaction, we further propose that the volcanic ash samples described here should be employed in future engine operation testing to assess the relative propensities of natural ash particles to stick and interact with the coating inside jet engines.

## Methods

### Sample characterization

Nine volcanic ash samples were collected from nine volcanoes around the world: Grímsvötn (Iceland, 24 May 2011); Fuego (Guatemala, 13 September 2012); Mount Merapi (Indonesia, 26 November 2010); Campi Flegrei (Italy, 15,000 years BP); Santa María (Guatemala, 28 November 2012); Karymsky (Russia, 7 May 2011); Unzen (Japan, 3 June 1991); Soufrière Hills (Montserrat, 10 February 2012); and Da'Ure (Ethiopia, 18 September 2005). We also acquired three standard materials used in ingestion tests: Arizona test dusts (ATD-A2 fine grade (<120 μm) and ATD-A4 coarse grade (<200 μm)) and military standard test sand (MIL E 5007C)^21^. To test whether the presence of crystals in natural volcanic ash affects the ash fusion behaviour in the melting process, three natural volcanic ash samples containing crystals (Campi Flegrei, Santa María and Soufrière Hills) were selected; for each sample ∼20 g of material was heated in platinum crucible to 1,650 °C and held for 48 h to ensure that all crystals melted. The melt was then quenched to glass by removing the crucible from the furnace and letting it to cool in air. The sample was then crushed and sieved to retrieve the same grain size as the natural ash. X-ray fluorescence (Philips Magix XRF spectrometer at 4 kV) and scanning electron microscope (JEOL JSM-5600) analyses were conducted on all samples to assess the bulk chemical composition as well as to observe the morphological changes of ash particles (Supplementary Table 1). Particle size distributions of milled ash were measured via a LS 230 laser diffraction particle analyser (Beckman Coulter).

### Characteristic temperature measurement

Each ash sample was first milled to generate particle finer than 63 μm, in accordance with the requirements of powder compacts' preparation for thermo-optical examination in a heating microscope (according to CEN/TS 15443 procedures). The ash sample was then compacted into 3-mm high and wide cylindrical cores by pressing the ash, with a pressure of 1.5 N mm^−2^ into cylindrical opening of a die, and the cylindrical ash compact was removed from the die by a de-moudling tool, onto an alumina substrate (99.7% Al_2_O_3_), which was carefully positioned on the sample holder and then put into the tube furnace. Owing to manual handling of the fragile ash compact, the ash compact may not stand fully upright and contact with the substrate may be partial.

Morphological changes of the compacts on heating were monitored in a heating microscope (Hesse Instruments), following standard requirements for the determination of ash melting and slagging behaviour. In this study, nine samples of natural volcanic ash, two of dusts, one of sand and three of remelted volcanic ash were heated to 1,650 °C at a rate of 10 °C min^−1^; for the ash sample from Santa María additional tests were conducted at 20, 30 and 40 °C min^−1^.

The fusion of ash is geometrically defined through four characteristic temperatures: ST, when the area of a sample core's silhouette shrinks by 5% of the original test piece area at 50 °C; DT, when the shape factor (defined in Supplementary Fig. 2) changes by 15%; HT, when the height becomes equal to half of the basal diameter; and FT, when the height of the spreading sample is one-third of the original test piece at 50 °C. Each sample was measured twice under the same conditions. All data for all experiments are exhibited in Supplementary Table 2.

### Thermomechanical assessment

To assess the thermomechanical properties of all investigated samples (including volcanic ash, dust and sand) at each characteristic temperature, we produced four ash compacts of each sample, which we successively heated to each one of the four previously determined characteristic temperatures in the heating microscope at a heating rate of 10 °C min^−1^. Once at a desired characteristic temperature the sample was immediately removed from the furnace to assess whether it stuck to the substrate by picking it up with steel tweezers. The samples retrieved from these tests arrested at each characteristic temperature were then sectioned for corresponding microstructure analysis using a scanning electron microscope.

### Water content measurement

The water (H_2_O) content in volcanic ash was constrained through thermogravimetric analysis (from Netzsch), which was performed in an air atmosphere with a flow rate of 25 ml min^−1^. For the measurements about 20–30 mg of the sample was weighed in a platinum crucible and heated up to 1,490 °C at a heating rate of 10 °C min^−1^. Weight change was monitored, which provides a proxy to estimate the magnitude of chemical reactions such as dehydration and oxidation. Weight loss at lower temperatures (below the glass transition temperature) is generally understood to result from the dehydration of secondary molecular water^37^. The water content was estimated by assessing the weight loss between the glass transition temperature (ca. 700 °C) and 1,200 °C, where water solubility is minimal^37^.

### Definition of a base–acid ratio

The base–acid ratio (*R*_b/a_) is a standard index to predict coal ash deposition behaviour in coal combustion, which compares the sums of weight fractions of basic and acidic oxides: base=CaO+FeO+MgO+K_2_O+Na_2_O+MnO; acid=SiO_2_+Al_2_O_3_+TiO_2_+ P_2_O_5_.

### Melting process analysis

We divided the range of melting processes of nine volcanic ash samples into three stages^12^, which are as follows: a shrinkage process (ST→DT) in which the area of all of volcanic ash sample compacts diminished significantly with temperature and time; a fusion process (DT→HT) in which a shape factor change increases with temperature and time, indicating the shapes' rounding, due to melting; and a wetting process (HT→FT) in which the basal contact's radius increases with temperature and time.

In the shrinkage process, the change of shrinkage was described with the parameter *S* (as the sample is simply and homogeneously reduced by 5% only at the onset of ST). The degree of shrinkage *S* can be calculated using:


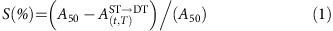


where *A*_50_ and 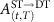 are the area of the a sample core's silhouette at 50 °C and the area after each interval time (*t*) or temperature (*T*) between ST and DT, respectively.

The average shrinkage rate in this stage, 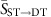, is defined as:


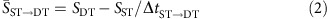


where *S*_DT_ and *S*_ST_ are the shrinkage at DT and ST, respectively. Δ*t*_ST→DT_ is the interval time between ST and DT (s).

For the fusion process, which begins as DT, where the initial change of shape factor being consistent with each sample, that is, 15% at DT, the degree of fusion *F* was defined by:


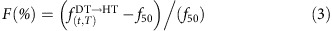


where *f*_50_ and 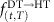
are the shape factor of the compact at 50 °C and the shape factor after each interval time (*t*) or temperature (*T*) between DT and HT, respectively.

The average fusion rate in this stage, 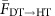, is defined as:


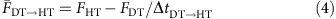


where *F*_HT_ and *F*_DT_ are the degree of fusion at the HT and DT, respectively. Δ*t*_DT→HT_ is the interval time between DT and HT (s).

Because the wetting process is a spontaneous spreading phenomenon of liquid droplets on solid substrate, we have chosen to track the change in basal contact diameter between melting volcanic ash and alumina oxide substrate to assess the spreading kinetics


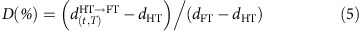


where *D* is the degree of wetting, and 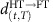, *d*_HT_ and *d*_FT_ are the diameter of the contact area of melted volcanic ash with substrate after each interval time (*t*) or temperature (*T*) in the range from HT to FT, and at HT and FT, respectively.

The average spreading velocity of wetting process in this stage was selected to describe the average wetting rate 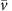, which is defined as:


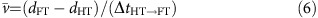


where 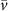 is average spreading velocity of wetting (mm s^−1^) and Δ*t*_HT→FT_ is the interval time between HT and FT (s).

### Viscosity calculation

The viscosity of the molten volcanic ash as a function of temperature was estimated using the bulk chemical composition of the ash as input parameter into the GRD silicate melt viscosity calculator^25^. On the basis of this viscosity model, the viscosity values corresponding to the characteristic temperatures for deposition (DT, HT and FT) of the volcanic ash samples were calculated.

## Additional information

**How to cite this article**: Song, W. *et al*. Volcanic ash melting under conditions relevant to ash turbine interactions. *Nat. Commun.* 7:10795 doi: 10.1038/ncomms10795 (2016).

## Supplementary Material

Supplementary Information Supplementary Figures 1-9 and Supplementary Tables 1-2.

Supplementary Data 1 This file contains the absolute and relative parameter values for describing dimensions and geometrical shape of the nine volcanic ash samples, two dusts, one sand and three remelted ash samples. These parameters includes the absolute value of area, height, baseline and shape factor as well as the ones used for determining the four characteristic temperatures including the change of area, shape factor and height as well as the ratio of height to baseline versus time and temperature in two independent experiments. Furthermore, the four characteristic temperatures of these samples are also marked in this file.

Supplementary Movie 1 This video shows the shape and dimension change of volcanic ashes (I-III columns), dust and sand (IV column) with increasing temperature and time at a rate of 10^°^C min^-1^.

## References

[b1] Sanderson K. Questions fly over ash-cloud models. Nature 464, 1253 (2010). 20428131 10.1038/4641253a

[b2] Tupper A. . Facing the challenges of the international airways volcano watch: the 2004/05 eruptions of Manam, Papua New Guinea. Wea. Forecasting 22, 175–191 (2007).

[b3] Prata A. J., Barton I. J., Johnson R. W., Kamo K. & Kingwell J. Hazard from volcanic ash. Nature 354, 25 (1991). 1944566 10.1038/354025a0

[b4] Dunn M. G., Padova C., Moller J. E. & Adams R. M. Performance deterioration of a turbofan and a turbojet engine upon exposure to a dust environment. J. Eng. Gas Turbines Power 109, 336–343 (1987).

[b5] Rose W. I. Interaction of aircraft and explosive eruption clouds–a volcanologist's perspective. AIAA J. 25, 52–58 (1987).

[b6] Hamed A., Tabakoff W. C. & Wenglarz R. V. Erosion and deposition in turbomachinery. J. Propul. Power 22, 350–360 (2006).

[b7] Abelson P. H. Jet-powered flight. Science 254, 497 (1991). 17806955 10.1126/science.254.5031.497

[b8] Padture N. P., Gell M. & Jordan E. H. Thermal barrier coatings for gas-turbine engine applications. Science 296, 280–284 (2002). 11951028 10.1126/science.1068609

[b9] Perepezko J. H. The hotter the engine, the better. Science 326, 1068–1069 (2009). 19965415 10.1126/science.1179327

[b10] Drexler J. M. . Jet engine coatings for resisting volcanic ash damage. Adv. Mater. 23, 2419–2424 (2011). 21484889 10.1002/adma.201004783

[b11] Casadevall T. J. Volcanic hazards and aviation safety—lessons of the past decade. FAA Aviation Safety J 2, 9–17 (1992).

[b12] Prata A. J. & Tupper A. Aviation hazards from volcanoes: the state of the science. Nat. Hazards 186, 91–107 (2009).

[b13] Schulz U. & Braue W. Degradation of La_2_Zr_2_O_7_ and other novel EB-PVD thermal barrier coatings by CMAS (CaO-MgO-Al_2_O_3_-SiO_2_) and volcanic ash deposits. Surf. Coat. Tech. 235, 165–173 (2013).

[b14] Carter T. J. Common failures in gas turbine blades. Eng. Fail. Anal. 12, 237–247 (2005).

[b15] Weinzierl B. . On the visibility of airborne volcanic ash and mineral dust from the pilot's perspective in flight. Phys. Chem. Earth A 45–46, 87–102 (2012).

[b16] Dunn M. G. Operation of gas turbine engines in an environment contaminated with volcanic ash. J. Turbomach. 134, 051001 (2012).

[b17] Wellman R. G. & Nicholls J. R. A review of the erosion of thermal barrier coatings. J. Phys. D Appl. Phys. 40, R293–R305 (2007).

[b18] Grant G. & Tabakoff W. Erosion prediction in turbomachinery resulting from environmental solid particles. J. Aircraft 12, 471–478 (1975).

[b19] Cardwell N. D., Thole K. A. & Burd S. W. Investigation of sand blocking within impingement and film-cooling holes. J. Turbomach. 132, 021020 (2010).

[b20] Batcho P. F., Moller J. C., Padova C. & Dunn M. G. Interpretation of gas turbine response due to dust ingestion. J. Eng. Gas Turbine Power 109, 344–352 (1987).

[b21] Kueppers U. . The thermal stability of Eyjafjallajökull ash versus turbine ingestion test sands. J. Appl. Volcanol. 3, 4 (2014).

[b22] Gertisser R. Eyjafjallajökull volcano causes widespread disruption to European air traffic. Geol. Today 26, 94–95 (2010).

[b23] Dingwell D. B., Lavallée Y. & Kueppers U. Volcanic ash: a primary agent in the earth system. Phys. Chem. Earth 45–46, 2–4 (2012).

[b24] White J. D. L. & Houghton B. F. Primary volcaniclastic rocks. Geology 34, 677–680 (2006).

[b25] Giordano D., Russell J. K. & Dingwell D. B. Viscosity of magmatic liquids: a model. Earth Planet. Sci. Lett. 271, 123–134 (2008).

[b26] Vasseur J. . Volcanic sintering: timescales of viscous densification and strength recovery. Geophys. Res. Lett. 40, 5658–5664 (2013). 25821262 10.1002/2013GL058105 PMC4373153

[b27] Wadsworth F. B. . Nonisothermal viscous sintering of volcanic ash. Solid Earth 119, 8792–8804 (2014).

[b28] D'Oriano C. . Effects of experimental reheating at different temperatures and redox conditions of natural basaltic ash. Contrib. Mineral. Petrol. 165, 863–883 (2013).

[b29] Ghiorso M. S. . Algorithms for the estimation of phase stability in heterogeneous thermodynamic systems. Geochim. Cosmochim. Acta 58, 5489–5501 (1994).

[b30] Gottsmann J., Giordano D. & Dingwell D. B. Predicting shear viscosity during volcanic processes at the glass transition: a calorimetric calibration. Earth Planet. Sci. Lett. 198, 417–427 (2002).

[b31] Song W. . Fusion characteristics of volcanic ash relevant to aviation hazards. Geophys. Res. Lett. 41, 2326–2333 (2014).

[b32] Mechnich P., Braue W. & Schulz U. High-temperature corrosion of EB-PVD yttria partially stabilized zirconia thermal barrier coatings with an artificial volcanic ash overlay. J. Am. Ceram. Soc. 94, 925–931 (2011).

[b33] Dunn M. G., Baran A. J. & Miatech J. Operation of gas turbine engine in volcanic ash clouds. J. Eng. Gas Turbines Power 118, 724–731 (1996).

[b34] Winegartner E. C. & Rhodes B. T. An empirical study of the relation of chemical properties to ash fusion temperatures. J. Eng. Gas Turbines Power 97, 395–403 (1975).

[b35] Ndamka N. L. *Microstructural Damage of Thermal Barrier Coatings due to CMAS* (PhD thesis, Univ. Cambridge, 2013).

[b36] Mysen B. O., Virgo D. & Seifert F. A. The structure of silicate melts: implications for chemical and physical properties of natural magma. Rev. Geophys. 20, 353–383 (1982).

[b37] Holtz F., Behrens H., Dingwell D. B. & Johannes W. H_2_O solubility in haplogranitic melts–compositional, pressure and temperature-dependence. Am. Mineral. 80, 94–108 (1995).

[b38] Denton J. S., Tuffen H., Gilbert J. S. & Odling N. The hydration and alteration of perlite and rhyolite from Iceland. J. Geol. Soc. Lond. 166, 895–904 (2009).

[b39] Hess K.-U. & Dingwell D. B. Viscosities of hydrous leucogranitic melts: a non-Arrhenian model. Am. Mineral. 81, 1297–1300 (1996).

[b40] Strangman T., Raybould D., Jameel A. & Baker W. Damage mechanisms, life prediction, and development of EB-PVD thermal barrier coatings for turbine airfoils. Surf. Coat. Technol. 202, 658–664 (2007).

[b41] Spitsberg I. & Steibel J. Thermal and environmental barrier coatings for SiC/SiC CMCs in aircraft engine applications. Int. J. Appl. Ceram. Technol. 1, 291–301 (2004).

[b42] Zhao H., Levi C. G. & Wadley H. N. G. Molten silicate interactions with thermal barrier coatings. Surf. Coat. Technol. 251, 74–86 (2014).

[b43] Kim J., Dunn M. G., Baran A. J., Wade D. P. & Tremba E. L. Deposition of volcanic materials in hot sections of two gas turbine engines. J. Eng. Gas Turbines Power 115, 641–651 (1993).

[b44] Casadevall T. J. The 1989-1990 eruption of Redoubt Volcano, Alaska: impacts on aircraft operations. J. Volcanol. Geotherm. Res. 62, 301–316 (1994).

[b45] Clarke D. R., Oechsner M. & Padture N. P. Thermal-barrier coatings for more efficient gas turbine engines. MRS Bull. 37, 891–897 (2012).

[b46] Sampath S., Schulz U., Jarligo O. M. & Kuroda S. Processing science of advanced thermal-barrier system. MRS Bull. 37, 903–910 (2012).

[b47] Mechnich P. & Braue W. Volcanic ash-induced decomposition of EB-PVD Gd_2_Zr_2_O_7_ thermal barrier coatings to Gd-Oxyapatite, Zircon, and Gd, Fe-Zirconolite. J. Am. Ceram. Soc. 96, 1958–1965 (2013).

