# Supplementary material for: Volcanic ash melting under conditions relevant to ash turbine interactions
Source: Nat Commun. 2016 Mar 2;7:10795. doi: 10.1038/ncomms10795 (PMC4778063; doi:10.1038/ncomms10795)
Supplement: Supplementary Information — Supplementary Figures 1-9 and Supplementary Tables 1-2. [file ncomms10795-s1.pdf]

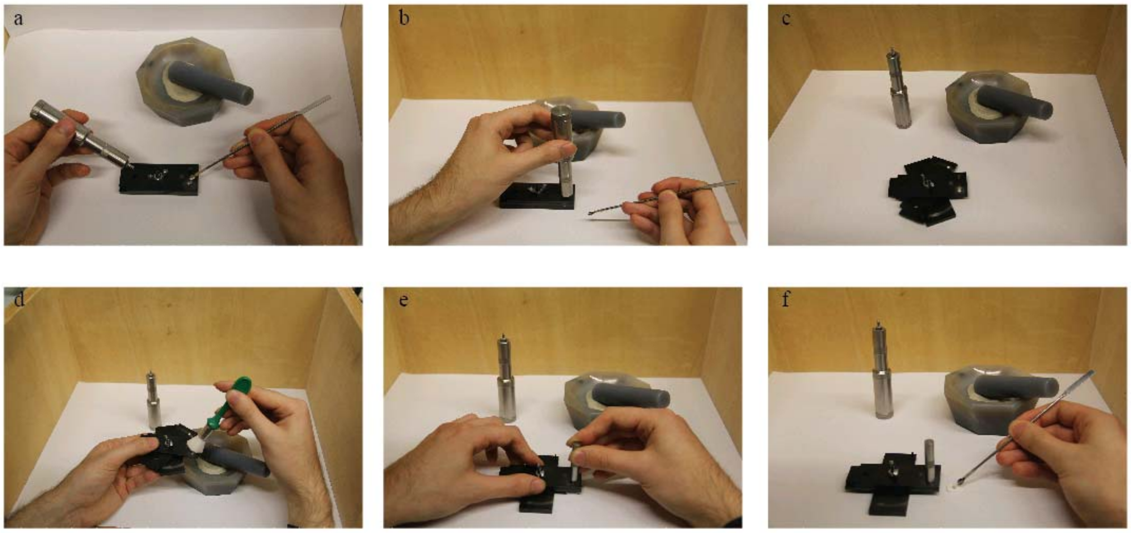

Supplementary Figure 1: Preparation of ash compact using a die. (**a**) Sample preparation; (**b**) Pressing of ash sample into die; (**c**) unlocking of the die; (**d**) removal of the ash compact. (**e-f**) placing the ash compact onto the alumina substrate using the small de-molding tool.

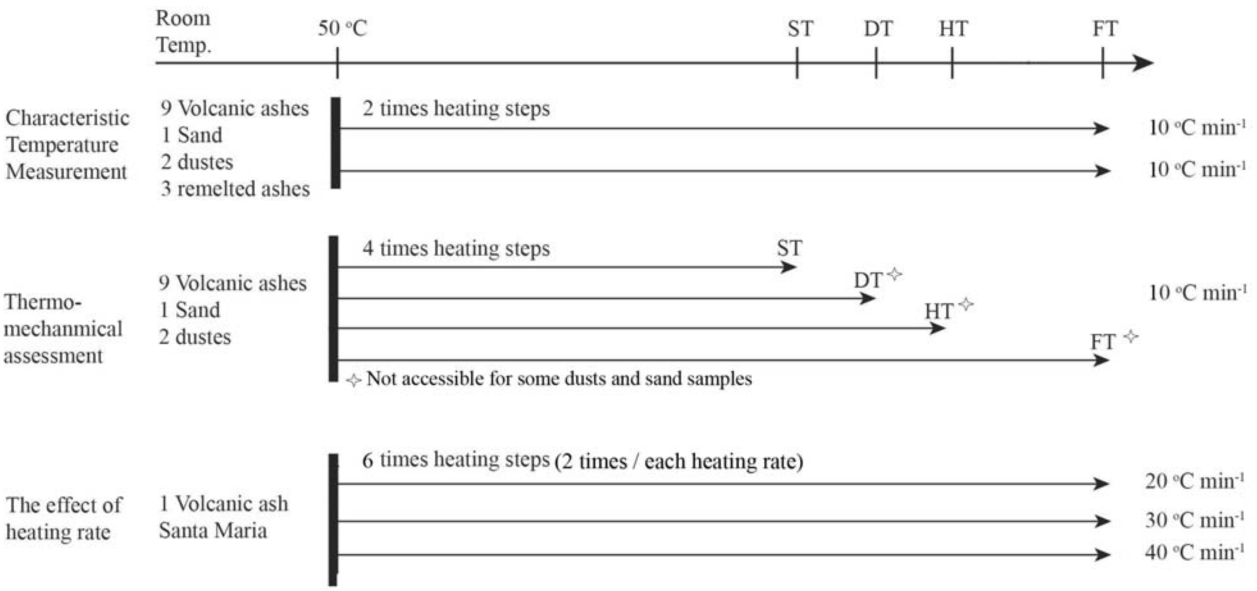

Supplementary Figure 2: Experimental schedule. We produced 6 ash compacts for each one of the 9 volcanic ash samples, 2 dust samples and 1 sand sample; we also produced 2 ash compacts for 3 remelted ash samples. The experiments were divided into three phases for different purposes. First, 2 of the 6 compacts of natural samples as well as the 6 remelted ash compacts were measured up to 1650 °C to determine the characteristic temperatures of each samples. Second, 4 of the 6 compacts were individually heated to each one of the characteristic temperatures to asses the sticking ability and to prepare thin sections for optical and SEM analyses. Third, 6 compacts of Santa María ash were measured at different heating rates.

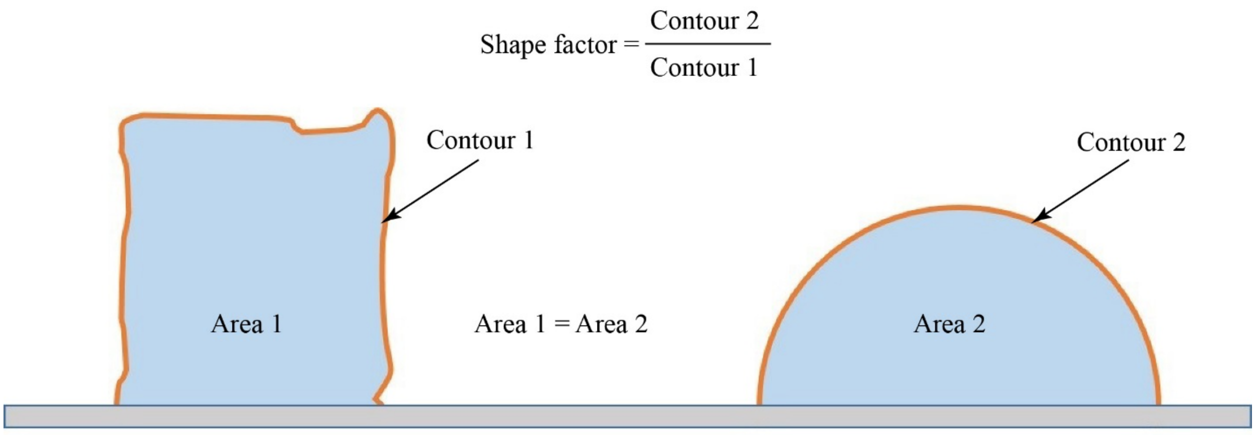

Supplementary Figure 3: Definition of shape factor. The shape factor is calculated via a ratio comparing the contour of a perfect semicircle (contour 2) with the contour of the initial specimen silhouettes (contour 2) in the heating microscope; thus: Shape factor = Contour 2 / Contour 1). (Diagram adapted from Hesse Instruments method description for heating microscope).

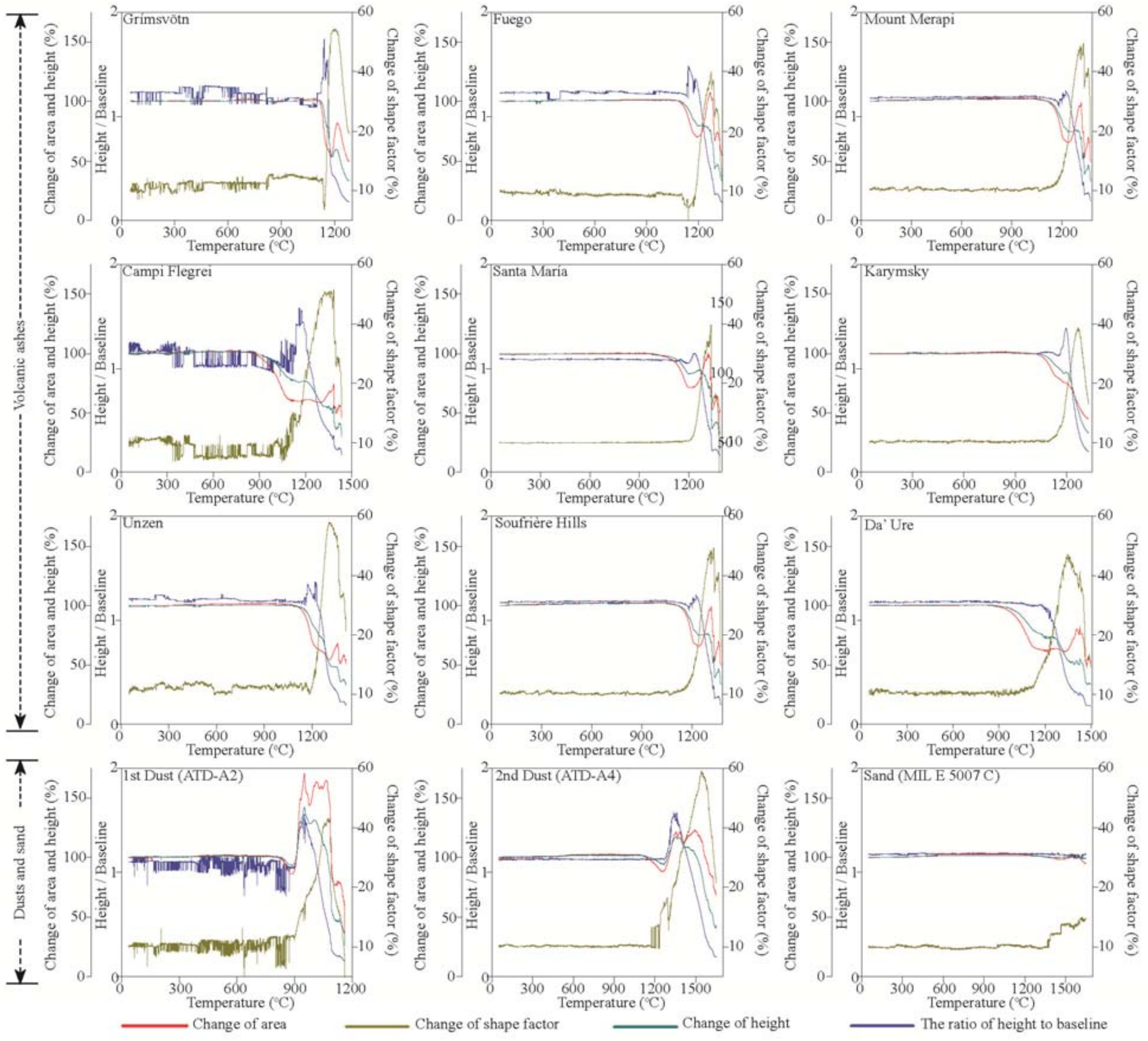

Supplementary Figure 4: Thermo-optical analysis of volcanic ash samples, dusts and sand. Geometrical evolution of the powder compacts' silhouette from microscope image analysis during testing at a heating rate of 10 °C min^{-1} as shown in Supplementary Movie 1. The change of area (red, ST), the shape factor (dark yellow, DT) and height (dark cyan, HT) as well as the ratio of height to base width (blue, FT) were plotted versus temperature. Data analysis of characteristic temperatures populated Supplementary Table 2.

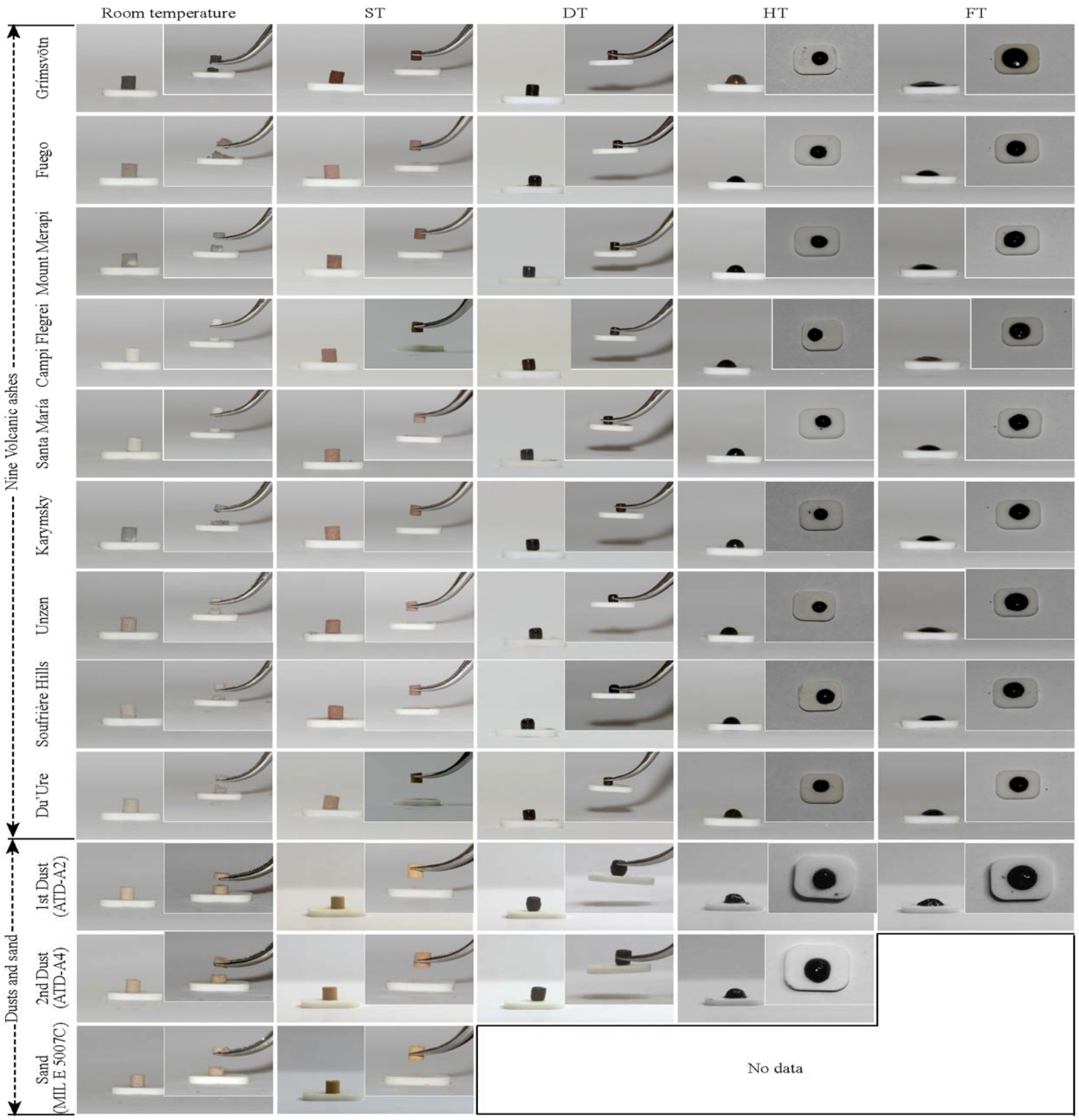

Supplementary Figure 5: Physical aspect of volcanic ash samples, dusts and sand upon fusion. Photographs showing powder compacts at the characteristic temperatures. Initially (column I), the powder compacts are too soft to be picked up with tweezers; at ST (column II), the powder compacts become coherent and harden. The samples can be picked with tweezers as they did not stick to the substrate; at DT (column III), the sample has stuck to the substrate; at the subsequent HT (column IV) and FT (column V), the sample spread on the substrate, showing the wetting and flow ability, respectively. Note that the blank space at the bottom right corner of the matrix refers to fact that the maximum tested temperature (1650 °C) was insufficient to reach these characteristic temperatures for the samples.

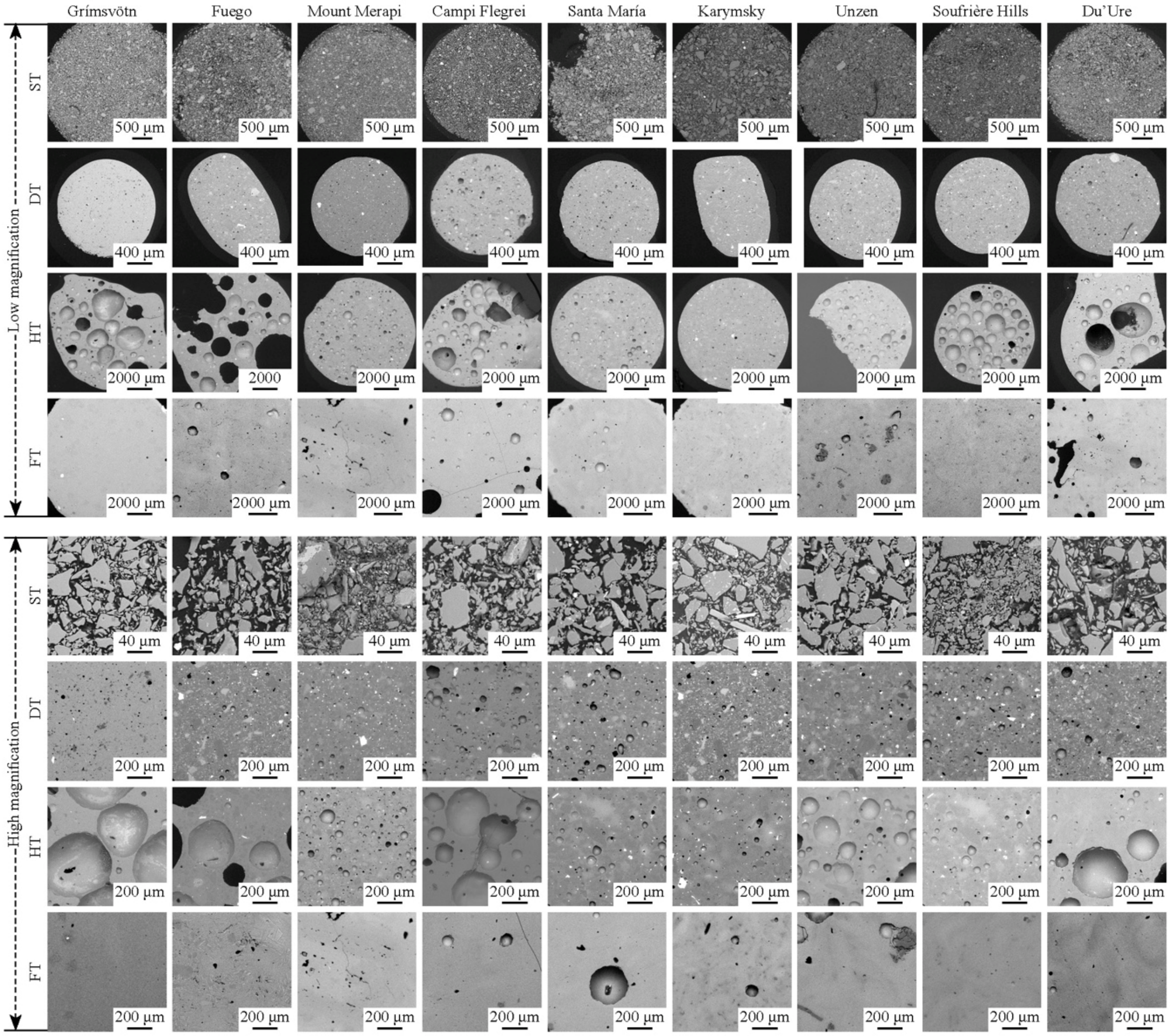

Supplementary Figure 6: Microstructural evolution of volcanic ash samples. The lower (top) and higher (bottom) magnification SEM images show the microstructures of studied volcanic ash samples at four characteristic temperatures (see Methods). At ST, the particles forming the compact remain distinguishable. At DT, the fragments are fully sintered and individual particles contours are no longer detectable; the sample contains some suspended pores. This transformation from particulate to coherent body reflects the transition from solid to melt, which corresponds to the onset of sticking. At HT the volume of isolated bubbles increases due to vesiculation, thermally-driven gas expansion and bubble coalescence. At FT, the amount of bubbles has significantly reduced due to outgassing.

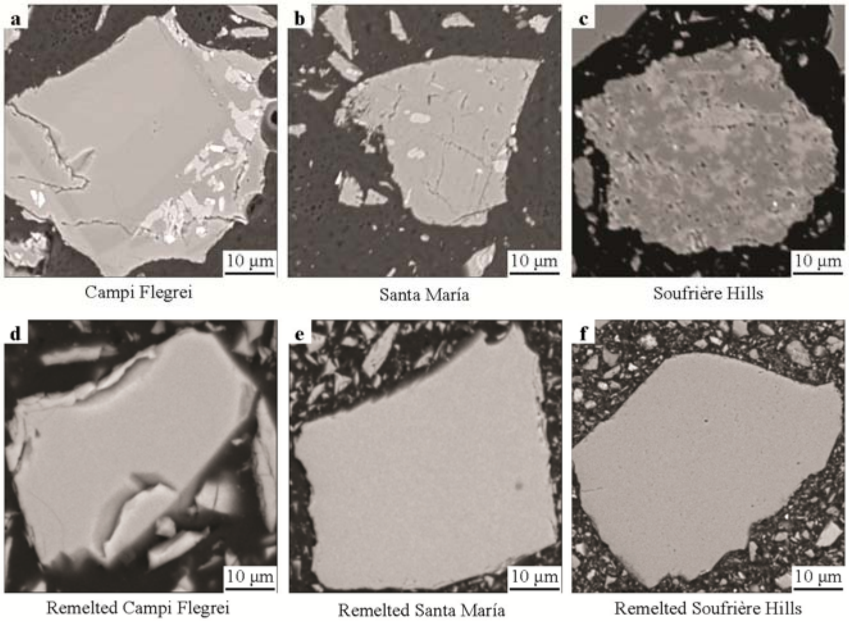

Supplementary Figure 7: Textural characteristic of natural ash and remelted ash particles. Example of a particle of natural volcanic ash samples from (**a**) Campi Flegrei, (**b**) Santa María, and (c) Soufrière Hills, consisting of glass and crystals. Example of a particle of remelted volcanic ash samples from (**d**) Campi Flegrei, (**e**) Santa María, and (**f**) Soufrière Hills which consist of homogenous glass fragments.

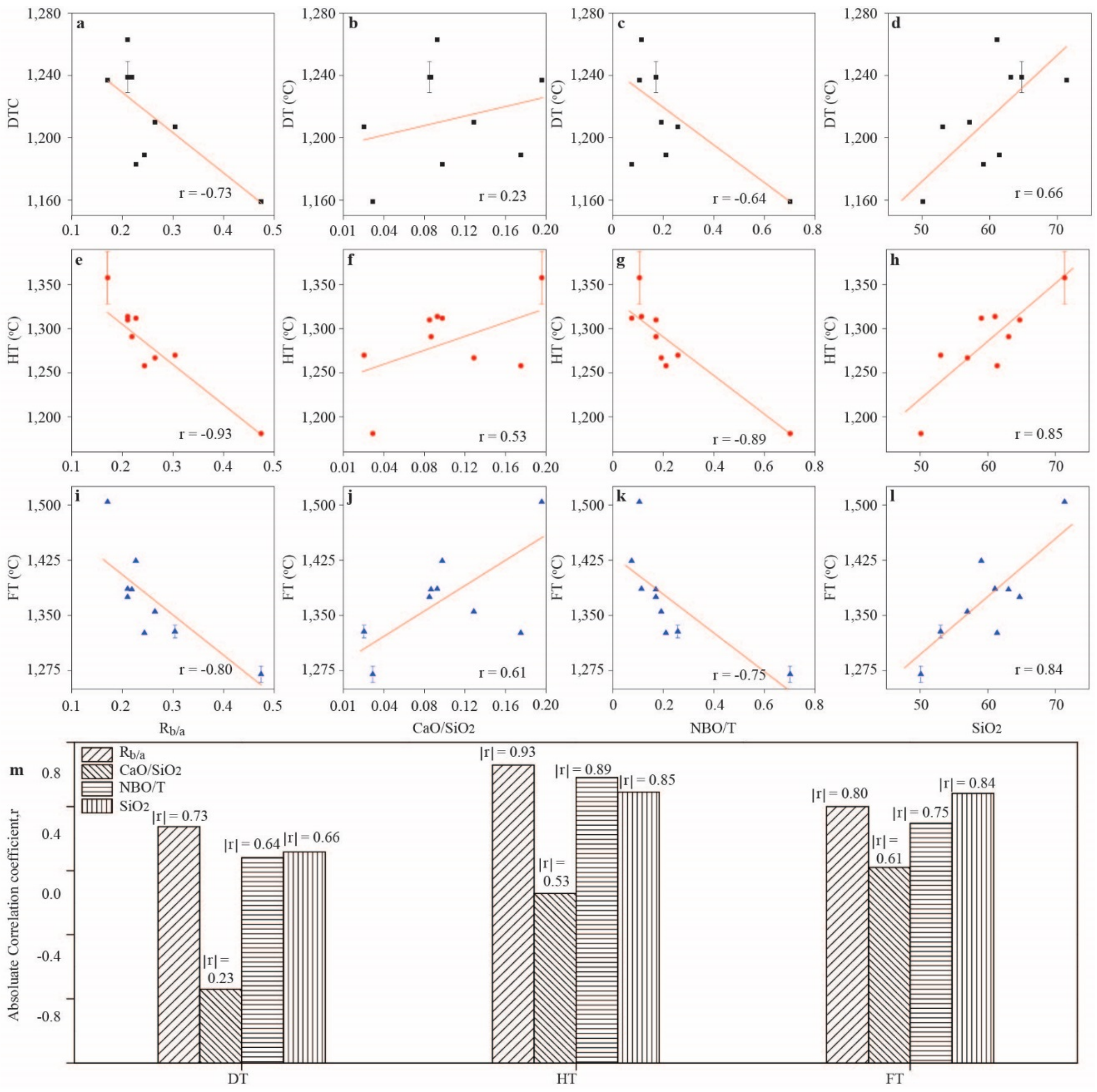

Supplementary Figure 8: Chemical effects on the fusion of volcanic ash samples. The diagrams report the characteristic temperatures (DT, HT and FT) against chemical composition as constrained by the metrics (**a**, **e**, **i**) $R_{b/a}$, (**b**, **f**, **j**) basicity index, CaO/SiO_2, (**c**, **g**, **k**) number of non-bridging oxygen per tetrahedron (NBO/T), and (**d**, **h**, **l**) SiO_2. The red lines are the respective linear regressions along with their correlation coefficient (r). (**n**) Bar plot showing the absolute correlation coefficient $|r|$ of the four metrics at DT, HT, and FT. The values represent the means with standard deviations of two independent experiments.

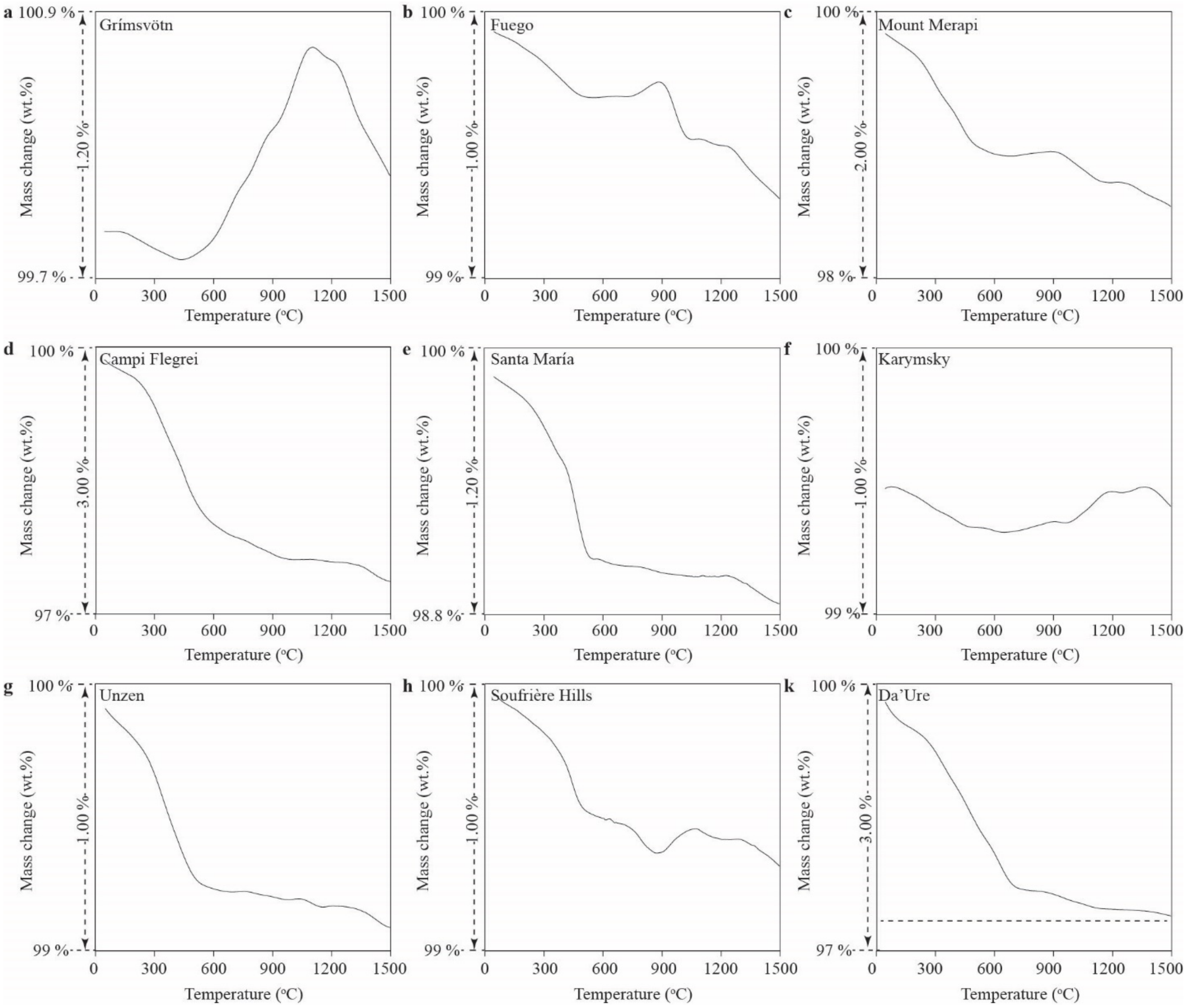

Supplementary Figure 9: Thermogravimetric (TG) analysis of volcanic ash samples. The TG curves display the weight change as temperature increases to 1490 °C at a rate of 10 °C min^{-1}.

Supplementary Table 1: Chemical and physical characteristics of the samples tested. The chemical composition is constrained by the the major oxides content (wt.%) and the *$R_{b/a}$* of the nine volcanic ash samples, two dusts, one sand and three remelted ash samples. The particle size distribution of these samples is also provided on the right-hand side.

Ash samples	Chemical composition of major oxides (wt.%)										R_b/R_a	Particle size		
	SiO_2	Al_2O_3	FeO	CaO	K_2O	Na_2O	TiO_2	MgO	MnO	P_2O_5		D_{10}	D_{50}	D_{90}
Grímsvötn (Iceland)	50.10	14.49	13.53	9.81	0.53	2.88	2.90	5.19	0.21	0.33	0.47	2.66	17.18	52.62
Fuego (Guatemala)	53.02	22.65	6.49	9.29	0.89	4.00	0.82	2.51	0.13	0.20	0.30	2.43	15.65	47.94
Mount Merapi (Indonesia)	56.99	21.14	5.30	7.34	2.48	3.94	0.59	1.71	0.15	0.35	0.26	2.21	10.78	33.01
Campi Flegrei (Italy)	59.05	21.85	2.60	1.71	5.68	7.75	0.48	0.48	0.27	0.14	0.23	1.67	17.18	57.77
Santa María (Guatemala)	61.07	20.83	3.94	5.96	1.44	4.58	0.49	1.37	0.11	0.22	0.21	3.52	22.73	57.77
Karymsky (Russia)	61.38	17.85	5.91	5.69	1.49	4.46	0.92	1.90	0.15	0.26	0.24	1.26	8.94	43.67
Unzen (Japan)	63.08	18.16	4.25	5.46	2.08	3.68	0.64	2.39	0.10	0.15	0.22	3.21	20.71	57.77
Soufrière Hills (Montserrat)	64.73	17.24	5.04	5.50	1.12	3.52	0.50	2.08	0.14	0.13	0.21	1.15	7.42	30.07
Da'Ure (Ethiopia)	71.38	13.62	3.63	1.46	4.31	4.56	0.37	0.55	0.10	0.02	0.17	3.21	22.73	57.77
1st Dust (ATD-A2)	76.96	11.22	3.08	2.59	2.76	1.37	0.40	1.39	0.09	0.14	0.12	1.26	8.15	18.86
2nd Dust (ATD-A4)	88.64	5.69	0.92	1.25	1.79	1.04	0.12	0.47	0.03	0.05	0.06	4.24	22.73	39.78
Sand (MIL E 5007 C)	94.48	2.93	0.45	0.04	1.67	0.22	0.13	0.15	0.00	0.03	0.03	2.01	14.26	30.07
[illegible]	58.91	22.96	2.93	1.75	5.08	6.51	0.96	0.46	0.22	0.22	0.20	3.21	20.71	57.77
[illegible]	62.15	19.64	3.99	5.99	1.46	4.49	0.51	1.41	0.12	0.23	0.21	1.15	7.42	30.77
[illegible]	65.74	16.00	5.28	5.57	1.06	3.38	0.52	2.12	0.14	0.17	0.21	3.21	22.73	57.77

Supplementary Table 2: Characteristic temperatures of nine volcanic ash samples, two dusts, one sand and three remelted volcanic ash samples. The data is provided for two runs and the average is calculated along with the standard deviation.

Ash samples	Heating rate (°C min^{-1})	Time	Realistic value				Standard deviation			
			ST	DT	HT	FT	ST	DT	HT	FT
Grímsvötn (Iceland)	10	1st	1125	1156	1181	1281	3.54	3.54	0.71	15.56
		2nd	1130	1161	1182	1259				
		Mean	1128	1159	1181	1270				
Fuego (Guatemala)	10	1st	1120	1210	1269	1336	2.83	4.95	0.71	12.02
		2nd	1124	1203	1270	1319				
		Mean	1122	1207	1270	1328				
Mount Merapi (Indonesia)	10	1st	1099	1209	1265	1352	2.12	0.71	2.83	4.24
		2nd	1102	1210	1269	1358				
		Mean	1101	1210	1267	1355				
Campi Flegrei (Italy)	10	1st	924	1182	1305	1432	4.24	0.71	9.19	12.02
		2nd	918	1183	1318	1415				
		Mean	921	1183	1312	1424				
Santa María (Guatemala)	10	1st	1098	1264	1312	1389	5.66	2.12	2.83	4.24
		2nd	1106	1261	1316	1383				
		Mean	1102	1263	1314	1386				
	20	1st	1102	1283	1340	1430	2.83	2.83	4.25	7.07
		2nd	1098	1287	1334	1420				
		Mean	1100	1285	1337	1425				
	30	1st	1086	1300	1356	1442	4.95	4.95	1.41	8.49
		2nd	1079	1305	1354	1430				
		Mean	1083	1303	1355	1436				
	40	1st	1044	1325	1364	1470	37.47	13.44	1.41	4.24
		2nd	1097	1306	1366	1464				
		Mean	1071	1316	1365	1467				
Karymsky (Russia)	10	1st	1096	1189	1258	1330	0	0.71	0	6.36
		2nd	1096	1188	1258	1321				
		Mean	1096	1189	1258	1326				
Unzen (Japan)	10	1st	1145	1239	1290	1382	0.71	0.71	0.71	3.54
		2nd	1144	1238	1291	1387				
		Mean	1145	1239	1291	1385				
Soufrière Hills (Montserrat)	10	1st	1153	1231	1310	1371	1.41	11.31	0	4.95
		2nd	1151	1247	1310	1378				
		Mean	1152	1239	1310	1375				
Da'Ure (Ethiopia)	10	1st	972	1231	1328	1498	0.71	8.49	41.72	7.78
		2nd	971	1243	1387	1509				
		Mean	972	1237	1358	1504				

#

Ash samples	Heating rate (°C min^{-1})	Time	Realistic value				Standard deviation			
			ST	DT	HT	FT	ST	DT	HT	FT
		1st	1133	1274	1428	1548				
1st Dust (ATD-A2)	10	2nd	1132	1278	1428	1544	0.71	2.83	0	2.83
		Mean	1133	1276	1428	1546				
		1st	1194	1319	1553	>1650				
2nd Dust (ATD-A4)	10	2nd	1189	1351	1552	>1650	3.53	22.63	0.71	2.83
		Mean	1192	1335	1553	>1650				
		1st	1642	>1650	>1650	>1650				
Sand (MIL E 5007 C)	10	2nd	>1650	>1650	>1650	>1650				
		Mean	>1650	>1650	>1650	>1650				
		1st	840	1165	1292	1451				
Remelted Campi Flegrei	10	2nd	844	1172	1315	1448	2.83	4.95	16.27	2.12
		Mean	842	1169	1304	1450				
		1st	869	1264	1304	1372				
Remelted Santa María	10	2nd	866	1262	1303	1366	2.12	1.41	0.71	4.24
		Mean	868	1263	1304	1369				
		1st	895	1218	1304	1399				
Remelted Montserrat	10	2nd	900	1230	1325	1399	3.54	8.49	14.85	0.00
		Mean	898	1224	1315	1399				
